# Sequential CT arterioportography-arteriosplenography depicts individual haemodynamic changes in children with portal hypertension without cirrhosis

**DOI:** 10.1186/s41747-020-00193-y

**Published:** 2020-12-02

**Authors:** Simone Hammer, Hans Jürgen Schlitt, Birgit Knoppke, Veronika Ingrid Huf, Walter Alexander Wohlgemuth, Wibke Uller

**Affiliations:** 1 Department of Radiology, University Hospital Regensburg, Franz-Josef-Strauss-Allee 11, 93053 Regensburg, Germany; 2 Department of Surgery, University Hospital Regensburg, Franz-Josef-Strauss-Allee 11, 93053 Regensburg, Germany; 3 KUNO University Children’s Hospital, University Hospital Regensburg, Franz-Josef-Strauss-Allee 11, 93053 Regensburg, Germany; 4 Department of Radiology, University Hospital Halle, Ernst-Grube-Str. 40, 06120 Halle, Germany

**Keywords:** Child, Hypertension (portal), Radiology (interventional), Portosystemic shunt (surgical), Tomography (x-ray computed)

## Abstract

We evaluated sequential computed tomography (CT) arterioportography-arteriosplenography for the assessment of venous pathways in children with portal hypertension without cirrhosis. Institutional Review Board approval was obtained for this retrospective, single-centre study. CT was performed after contrast application via catheters placed in the superior mesenteric artery (CT arterioportography) and the splenic artery (CT arteriosplenography) consecutively. Venous pathways in 22 children were evaluated. In all patients, the detailed haemodynamic consequences of portal hypertension could be characterised. The supply of varices at different locations could be assigned to the superior mesenteric vein or splenic vein system. Retrograde blood flow through the splenic vein and inferior mesenteric vein, portosystemic shunting, and patency of splanchnic veins were determined. CT arterioportography-arteriosplenography allowed a complete evaluation of individual haemodynamic pathways in children with portal hypertension.

## Key points

- Computed tomography (CT) arterioportography was combined with CT arteriosplenography in one session.
- This technique showed a comprehensive delineation of abdominal venous changes including source of varices, direction of blood flow, and portosystemic shunts.
- Using this approach, planning of individual interventional or surgical management of children with portal hypertension may become more appropriate.

## Background

Portal hypertension (PH) results from increased pressure in the portal venous system. It is a frequent problem in adult patients with liver cirrhosis. In children, PH can develop from a variety of conditions, but is frequently associated with intra- or extrahepatic venous obstruction [1]. Consequences of PH in children are haemodynamic changes in veins in the entire abdomen that include portosystemic shunts, reversal of flow directions, and varices that can lead to gastrointestinal bleeding and, among others, to splenomegaly and thrombocytopenia, ascites, hepatic encephalopathy, and growth failure [2]. Accordingly, morbidity and mortality rates for paediatric PH are clinically relevant with gastrointestinal bleeding representing the major life-threatening complication [2–4]. Prevention and treatment of variceal bleeding includes pharmacological management, endoscopic, or radiologic interventions and shunt surgery but long-lasting therapeutical success is difficult to achieve [5, 6].

One major problem for therapy of PH in children is the high variability of venous pathways in individual patients [7]. The individual assessment of venous circulation in paediatric PH, however, is critical for an adequate therapeutic intervention. To date, no imaging modality has been available to detect portosystemic shunts including the direction of blood flow in all splanchnic veins as well as main supply of varices via the splenic vein (SV) or portal vein (PV) system in every abdominal location simultaneously.

We report herein a new method for the detection of haemodynamic venous changes in paediatric PH. It is based on computed tomography (CT) with sequential contrast application into the splenic artery (SA) and the superior mesenteric artery (SMA). This technique was refined in order to reduce any dilution of contrast media in the systemic circulation and to identify all abdominal venous haemodynamic changes of (1) the superior mesenteric vein (SMV) and (2) the SV system, separately, in particular for differentiating the main supply of varices and identifying blood flow direction.

The objective of this study was to determine feasibility of this innovative technique and to determine the ability to detect individual haemodynamic changes in a small sample of children with portal hypertension.

## Methods

This study was conducted according to the principles expressed in the Declaration of Helsinki. Institutional Review Board approval was obtained. This was a retrospective, single-centre case series of paediatric patients who underwent CT AP-AS at our tertiary referral university hospital centre between November 2011 and September 2018. Children (18 years old or younger) with PH who underwent CT AP-AS were included.

### CT AP-AS technique

The procedures were performed under general or local anaesthesia and intravenous sedation using a prospectively determined standard angiographic technique. Every patient received 100 IU of heparin per kilogramme body weight at the beginning of the intervention.

The SMA and SA were selectively catheterised by transfemoral approaches using 4-F catheters through 4-F sheaths. Catheters were advanced in the main branch of the SMA and the SA proximal to the first side branch. Two standard digital subtraction angiography studies were performed by separated manual contrast agent injection (Iomeprol; Imeron 300, 300 mg I/mL, Bracco Imaging SpA, Milan, Italy) in the SA and in the SMA. This was performed (1) to confirm the catheter tip position and (2) to determine the individual time to contrast appearance in the SV and PV or related collateral vessels. This individual time was measured starting from contrast application in the SMA/SA to appearance of contrast medium in the PV/SV using a timer. Afterwards, the patients were transferred to the CT suite with the catheters secured to the skin at the groin.

Sequential CT AP-AS was performed with a helical 256 (2 × 128)-slice dual source CT scanner (Somatom Flash scanner; Siemens, Forchheim, Germany) using a prospectively determined CT protocol with automatic tube voltage selection (80 kV or 100 kV) and automatic tube current modulation in caudocranial scan direction. First, 0.4 mL nonionic contrast agent (Iopromid; Ultravist 300, 300 mg I/mL, Bayer Healthcare, Berlin, Germany) was injected per kilogramme bodyweight diluted with the same volume of NaCl at a rate of 2.5 mL/s in the SMA. Scanning was initiated after that time period that was individually measured angiographically, with a CT AP scan delay equal to the time from contrast application in the SMA to appearance in the PV or collateral vessels in the angiogram *plus* 5 s due to the rate of contrast agent administration, to achieve maximal contrast appearance in the targeted veins. Then, the same procedure was analogously performed with contrast injection in the SA, with a CT AS scan delay equal to time from contrast application in the SA to appearance in the SV in the angiogram *plus* 5 s. For image analysis, transverse and coronal plane images with a section thickness of 2.0 mm and a section increment of 2.0 mm were reconstructed.

**Fig. 1:**
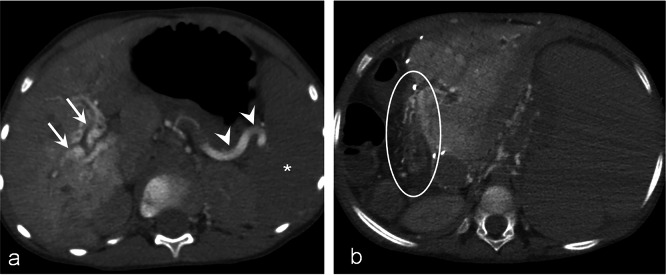
Computed tomography arterioportography (CT AP) findings in two different children. **a** A 5-year-old male patient with portal vein thrombosis. Axial images obtained after contrast injection in the superior mesenteric artery detect portal biliopathy (arrows) and reversed blood flow in the splenic vein (arrowheads). The spleen does not show contrast enhancement (white asterisk). **b** An 8-year-old male patient with portal vein thrombosis after liver transplantation. CT AP shows varices and diffuse enhancement of the hepaticojejunostomy (ellipse)

The CT AP-AS images of the 22 patients were evaluated in random order by two radiologists (with 12 and 14 years of experience in the interpretation of vascular CT studies) using a structured template by comparing the enhancement patterns of CT AP and AS, in consensus. Existence and site of varices and differentiation of their origin and main supply (SV system or SMV system) were documented. The presence and localisation of spontaneous portosystemic shunts were distinguished. Intra- and extrahepatic portal vein branches, SMV, and SV were reviewed for patency. Additionally, flow direction in the SV and inferior mesenteric vein (IMV) was determined by comparing the scans performed after contrast injection in the SMA and the SA in every patient. Retrograde blood flow in the SV and IMV was judged to be present in the case of enhancement of these veins after contrast injection into the SMA (Fig. 1a). Available standard CT in portal venous phase (performed less than 12 months before or after CT AP-AS without any PH treatment during this period) was compared to CT AP-AS using the same structured template. Readers were blinded to patients’ history and clinical data. Effective dose value was calculated by the vendor-specific software Teamplay Dose (Siemens, Forchheim, Germany). Numerical variables were summarised using descriptive statistics (median, minimum, and maximum). Categorical variables were reported as frequency and percentage.

## Results

Between November 2011 and September 2018, 25 children underwent CT AP-AS. Three patients without diagnosis of PH were excluded. Accordingly, the patient population consisted of 22 children with PH without cirrhosis who underwent CT AP-AS (median age 8.6 years, 2.6–17.4 years; median body weight 29.4 kg, 14.0–79.7 kg).

Fifteen children suffered from extrahepatic portal vein obstruction. The PH resulted in splenomegaly (*n* = 21), hypersplenism (*n* = 18), status post-gastrointestinal bleeding (*n* = 12), additional anaemia (*n* = 11), and ascites (*n* = 5). CT AP-AS was performed with technical success in all cases. The median scan delay was 20 s (14–28 s) for CT AP and 18 s (12–29 s) for CT AS, respectively. Median dose-length product for both CT scans was 338 mGy × cm (49–1128 mGy × cm). Median effective dose value was 7.5 mSv (1.1–17.1 mSv). No complications related to this technique occurred.

Oesophageal (*n* = 16), paraoesophageal (*n* = 18), gastric (*n* = 21), duodenal (*n* = 11), small intestinal (*n* = 11), colonic (*n* = 9), rectal (*n* = 6), peripancreatic (*n* = 7), umbilical (*n* = 2), and paraumbilical (*n* = 2) varices were detected. Varices located at the hepaticojejunostomy were detected in 3 of 4 of children who previously underwent liver transplantation (Fig. 1b). Peribiliar (*n* = 18) and gallbladder varices (*n* = 9) were identified.

**Fig. 2:**
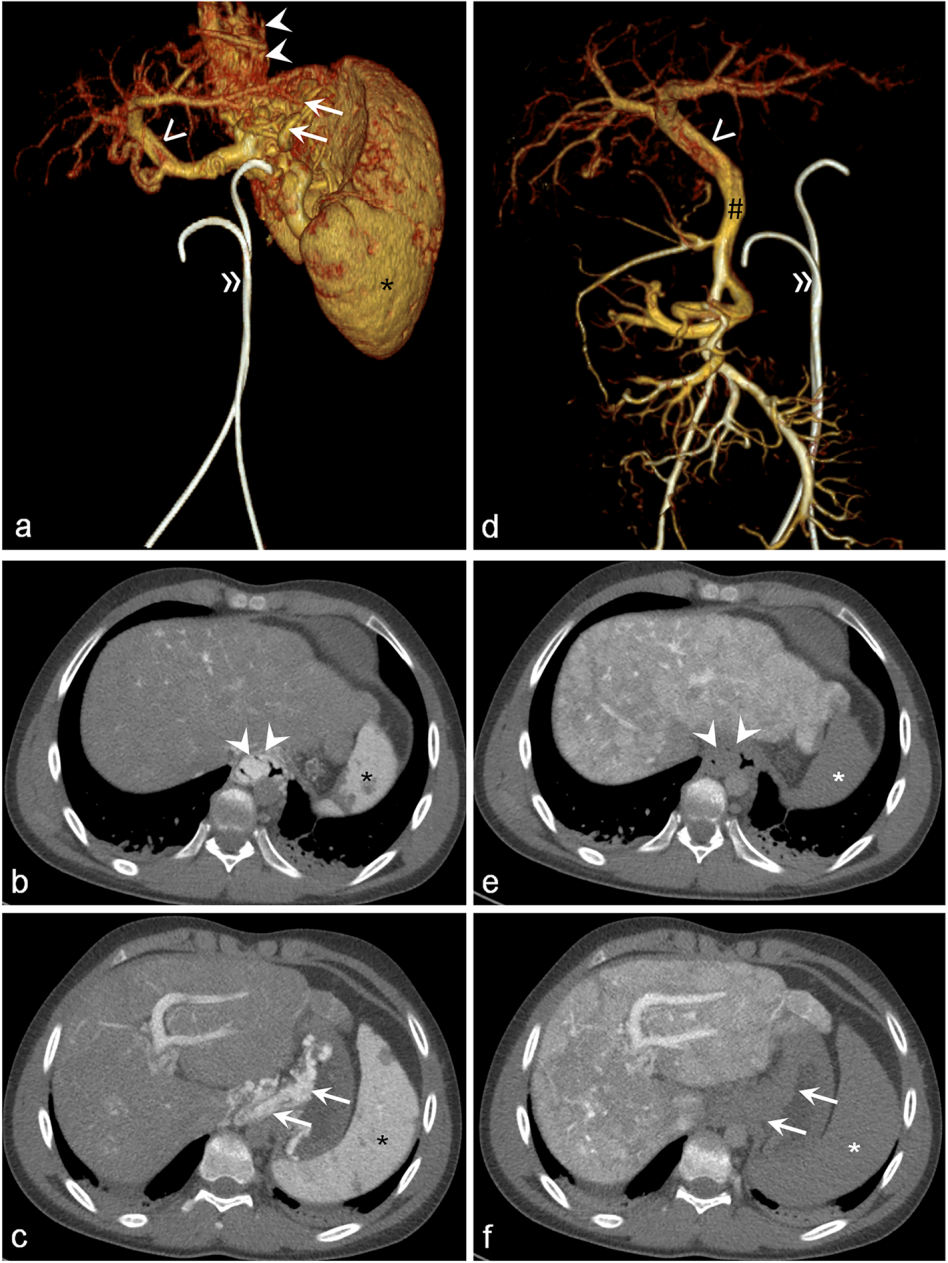
A 16-year-old male patient with portal hypertension. **a**–**c** Volume rendering and axial images of computed tomography arteriosplenography after contrast injection into the splenic artery allow to detect filling of oesophageal (**b**, arrowheads) and gastric (**c**, arrows) varices. Note the contrast enhancement of the spleen (black asterisk). **d**–**f** Volume rendering and axial images of computed tomography arterioportography after contrast injection into the superior mesenteric artery detect filling of the superior mesenteric vein (hashtag) and portal vein (open arrowhead) without any filling of oesophageal (**e**, arrowheads) or gastric varices (**f**, arrows). Note the absence of contrast enhancement of the spleen (white asterisk). Catheters placed in the superior mesenteric and splenic artery (double arrow). Portal vein (open arrowhead). Superior mesenteric vein (hashtag)

All varices located within the small intestine, hepaticojejunostomy, colon, and gallbladder were supplied by the SMV. The following varices were supplied by the SMV system, the SV system, or both (Fig. 2): oesophageal (6 SMV, 7 SV, 3 both), paraoesophageal (5 SMV, 12 SV, 1 both), gastric (7 SMV, 14 SV), duodenal (7 SMV, 3 SV, 1 both), peripancreatic (2 SMV, 2 SV, 3 both), and rectal varices (2 SMV, 4 SV).

Obstruction of the SMV (*n* = 2) and SV (*n* = 1) was found. The SMV-SV confluence was occluded in 6 cases. Occlusion of the extrahepatic PV (*n* = 15) and intrahepatic PV (*n* = 11) was detected resulting in extra- and intrahepatic cavernous transformation of the PV in 15 and 5 cases, respectively. Additionally, 16 children showed signs of portal biliopathy (multiple extrinsic/peribiliary collateral vessels, irregular and dilated bile ducts). Retrograde venous blood flow was seen in the SV in 14 cases and in the IMV in 8 cases. Splenorenal (*n* = 20) and additional shunts (vena haemiazygos, *n* = 10; inferior caval vein, *n* = 7) were found. Table 1 summarises the findings of CT AP-AS.

**Table 1:** Haemodynamic changes in 22 children with portal hypertension detected by sequential computed tomography arterioportography-arteriosplenography

Findings	Number of findings	Main venous supply of varices by		
		SMV	SV	SMV and SV
**Varices**				
Oesophageal	16	6	7	3
Paraoesophageal	18	5	12	1
Gastric	21	7	14	0
Duodenal	11	7	3	1
Small intestinal	11	11	0	0
Colonic	9	9	0	0
Rectal	6	2	4	0
Peripancreatic	7	2	2	3
Umbilical	2	2	0	0
Paraumbilical	2	0	0	2
Biliary	18	18	0	0
Portal biliopathy	16			
Gallbladder	9	9	0	0
Hepaticojejunostomy	3	3	0	0
**Portosystemic shunts**				
Splenorenal	20			
Shunt to haemiazygos	10			
Shunt to inferior caval vein	7			
**Occlusion of splanchnic veins**				
Extrahepatic portal vein	15			
Cavernous transformation	15			
Extrahepatic portal vein				
Intrahepatic portal vein	11			
Cavernous transformation	5			
Intrahepatic portal vein				
Extra- and intrahepatic portal vein	10			
SMV	2			
SV	1			
Confluence	6			
**Retrograde venous blood flow**				
SV	14			
Inferior mesenteric vein	8			

*SMV* Superior mesenteric vein, *SV* Splenic vein

**Fig. 3:**
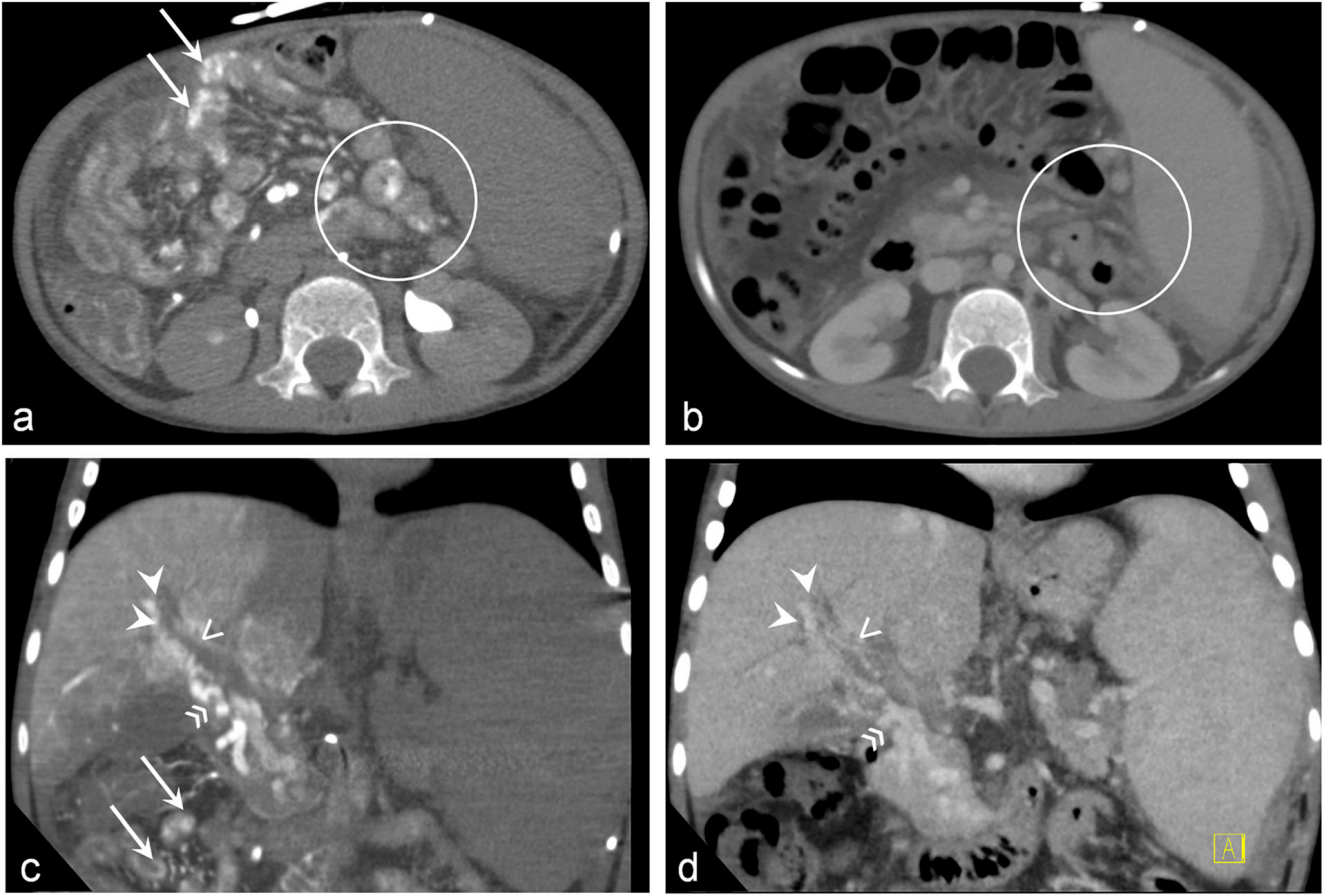
Comparison of computed tomography (CT) arterioportography (AP) (**a**, **c**) with standard CT after intravenous contrast injection (portal venous phase: **b**, **d**) in a 6-year-old male patient with portal vein thrombosis. Axial (**a**) and coronal (**c**) images of CT AP after contrast injection into the superior mesenteric artery allow to clearly detect multiple jejunal varices (arrows and circle) and portal biliopathy (arrowheads and open arrows: multiple small peribiliary collaterals, irregular and dilated bile ducts). Axial (**b**) and coronal (**d**) standard CT in the portal venous phase after intravenous contrast injection performed 2 weeks before CT AP does not detect any jejunal varices; evidence of portal biliopathy is lower than that provided by CT AP

In 5 cases, standard CT in portal venous phase was available. Oesophageal varices were detected in both, CT in portal venous phase and CT AP-AS equally. Of these 5 patients, CT in portal venous phase did not show any gastric, duodenal, small intestine, and colonic varices while CT AP-AS detected gastric varices in 5/5, duodenal varices in 2/5, small intestine varices in 3/5, and colonic varices in 3/5 patients (Fig. 3). Furthermore, CT AP-AS showed signs of portal biliopathy in 3 cases and a total of 10 portosystemic shunts, whereas none of these changes was detected by standard CT in portal venous phase. Extrahepatic and intrahepatic PV were occluded on standard CT in 2 and 3 cases, respectively, while CT AP-AS revealed patency of the extrahepatic and intrahepatic PV in these patients.

## Discussion

CT AP with contrast application in the SMA was initially performed to detect liver lesions [8–12]. Reinig et al. [13] used this technique for evaluating the anatomy of the PV system and the extent of PV thrombosis to facilitate accurate anatomical assessment prior to shunt surgery. This approach was also used for more accurate delineation of portosystemic shunts [14, 15]. However, contrast injection solely into the SMA has never been combined with CT AS to evaluate varices and their main venous supply as well as reversal blood flow, portosystemic shunts, and patency of splanchnic veins.

Using combined CT AP-AS, we were able to demonstrate a wide range of variable individual haemodynamic changes in children with portal hypertension. Venous routes and different tributaries varied considerably. Interestingly, the source of varices differed extremely and could be assigned to the SV or the SMV system. Additionally, even small varices at the hepaticojejunostomy in children after liver transplantation were identified as source of occult bleeding. Hence, this technique was also able to identify varices in localisations where endoscopy is not easily feasible or fails [16, 17]. Furthermore, our technique detected occlusion of splanchnic veins or changes in blood flow direction. Although the flow of the splanchnic veins can in principle also be detected using ultrasonography, in our experience, overlying structures often interfere with direct imaging of the SMV or SV in paediatric ultrasonography.

Following direct contrast injection into the SA and the SMA, no dilution effects from the systemic circulation occurred. This is critical for obtaining a strong contrast enhancement in the splanchnic veins and varices, allowing accurate anatomical and dynamical assessment. Compared to standard contrast-enhanced CT in portal venous phase, CT AP-AS proved to be superior not only in detecting the source of varices but also in the detection of different varices in other localisations than oesophageal. According to our results, CT AP-AS also proved patency of the extra- and intrahepatic PV, although standard contrast-enhanced CT did not show contrast enhancement in these vessels and simulated occlusions.

To our knowledge, combined CT AS-AP is the first technique that reveals every kind of haemodynamic venous changes in all abdominal locations in an objective, three-dimensional way and allows multiplanar reformatting. This comprehensive delineation of venous haemodynamic changes is of particular interest in complicated cases where the most appropriate management approach needs to be discussed and determined by interdisciplinary teams of interventional radiologists, surgeons, and gastroenterologists.

The major limitations of our study are that we did not have any comparative control group available and comparative imaging techniques as reference standard are missing.

In conclusion, although this study is additionally limited by its retrospective design and the small study cohort (based on the low incidence of PH without cirrhosis in children), our approach can help in detecting changes with consequences for individual treatment decisions. Important clinical points are as follows: (1) portosystemic shunts not visible on standard CT angiography may be a source of alternative access routes for interventional radiological treatment of varices (*e.g.*, via the femoral vein/renal vein/inferior of superior caval vein), (2) delineation of patency and reversal flow in the splanchnic veins is of particular interest prior to shunt surgery, and (3) the individual source of varices is of utmost interest in planning decompression of either the SV or the SMV system. No imaging modality was available to answer all these relevant questions in symptomatic paediatric PH simultaneously. However, all this information needs to be available in order to plan and adapt further management in every single case since indiscriminate interventions may result in deterioration of PH. CT AP-AS could be applied before more invasive procedures are considered. Future studies are necessary to address treatment outcome of individual management of complicated PH in children based on CT AP-AS findings.

## Data Availability

All relevant data is available within the manuscript.

## Abbreviations

AP: Arterioportography
AS: Arteriosplenography
CT: Computed tomography
IMV: Inferior mesenteric vein
PH: Portal hypertension
PV: Portal vein
SA: Splenic artery
SMA: Superior mesenteric artery
SMV: Superior mesenteric vein
SV: Splenic vein

## References

[1] Vogel CB (2017). Pediatric portal hypertension: a review for primary care. Nurse Pract.

[2] Grammatikopoulos T, McKiernan PJ, Dhawan A (2018). Portal hypertension and its management in children. Arch Dis Child.

[3] Shneider B, Emre S, Groszmann R (2006). Expert pediatric opinion on the Report of the Baveno IV consensus workshop on methodology of diagnosis and therapy in portal hypertension. Pediatr Transplant.

[4] Shneider BL, Bosch J, de Franchis R (2012). Portal hypertension in children: expert pediatric opinion on the report of the Baveno v Consensus Workshop on Methodology of Diagnosis and Therapy in Portal Hypertension. Pediatr Transplant.

[5] Cortez AR, Kassam A-F, Jenkins TM (2019). The role of surgical shunts in the treatment of pediatric portal hypertension. Surgery.

[6] Kassam A-F, Goddard GR, Johnston ME (2020). Natural course of pediatric portal hypertension. Hepatol Commun.

[7] Kirby JM, Cho KJ, Midia M (2013). Image-guided intervention in management of complications of portal hypertension: more than TIPS for success. Radiographics.

[8] Ferrucci JT (1990). Liver tumor imaging: current concepts. AJR Am J Roentgenol.

[9] Lupetin AR, Cammisa BA, Beckman I (1996). Spiral CT during arterial portography. Radiographics.

[10] Matsui O, Takashima T, Kadoya M (1985). Dynamic computed tomography during arterial portography: the most sensitive examination for small hepatocellular carcinomas. J Comput Assist Tomogr.

[11] Matsui O, Takashima T, Kadoya M (1987). Liver metastases from colorectal cancers: detection with CT during arterial portography. Radiology.

[12] Soyer P, Bluemke DA, Fishman EK (1994). CT during arterial portography for the preoperative evaluation of hepatic tumors: how, when, and why?. AJR Am J Roentgenol.

[13] Reinig JW, Sanchez FW, Vujic I (1985). Hemodynamics of portal blood flow shown by CT portography. Work in progress. Radiology.

[14] Ibukuro K, Tsukiyama T, Mori K, Inoue Y (1998) Veins of Retzius at CT during arterial portography: anatomy and clinical importance. Radiology 209:793–800. 10.1148/radiology.209.3.9844676 10.1148/radiology.209.3.9844676 9844676

[15] Terayama N, Matsui O, Kobayashi S (2008). Portosystemic shunt on CT during arterial portography: prevalence in patients with and without liver cirrhosis. Abdom Imaging.

[16] Nishimura N, Yamamoto H, Yano T (2010). Safety and efficacy of double-balloon enteroscopy in pediatric patients. Gastrointest Endosc.

[17] Sanada Y, Mizuta K, Yano T (2011). Double-balloon enteroscopy for bilioenteric anastomotic stricture after pediatric living donor liver transplantation. Transpl Int.

